# Ventricular Tachycardia Storm Induced by Loperamide Abuse

**DOI:** 10.7759/cureus.3981

**Published:** 2019-01-29

**Authors:** Jihad Al-khatib, Shravani R Vindhyal, Venkata S Boppana, Mohinder R Vindhyal

**Affiliations:** 1 Internal Medicine, University of Kansas School of Medicine, Wichita, USA; 2 Cardiology, University of Kansas School of Medicine, Wichita, USA

**Keywords:** ventricular tachycardia, loperamide, opioid crisis

## Abstract

A recent increase in the incidence of ventricular arrhythmias has been observed with the consumption of large dosages of the anti-diarrheal agent, loperamide. Our case is unique in that our patient not only displayed ventricular tachycardia (VT) but sustained VT (known as a VT storm). In this era of the opioid crisis, clinicians should be aware of all of the over-the-counter medications that have opioid-like side effects.

## Introduction

Loperamide is a commonly used and relatively safe anti-diarrheal agent that is available over-the-counter. The drug primarily works as an opioid receptor agonist within the gastrointestinal tract to decrease intestinal motility. In recent years, substance abusers have exploited the drug's agonizing opioid properties by ingesting large amounts of the drug to induce symptoms of euphoria. We present a case with a ventricular tachycardia storm following ingestion of large doses of loperamide. 

## Case presentation

**Figure 1:**
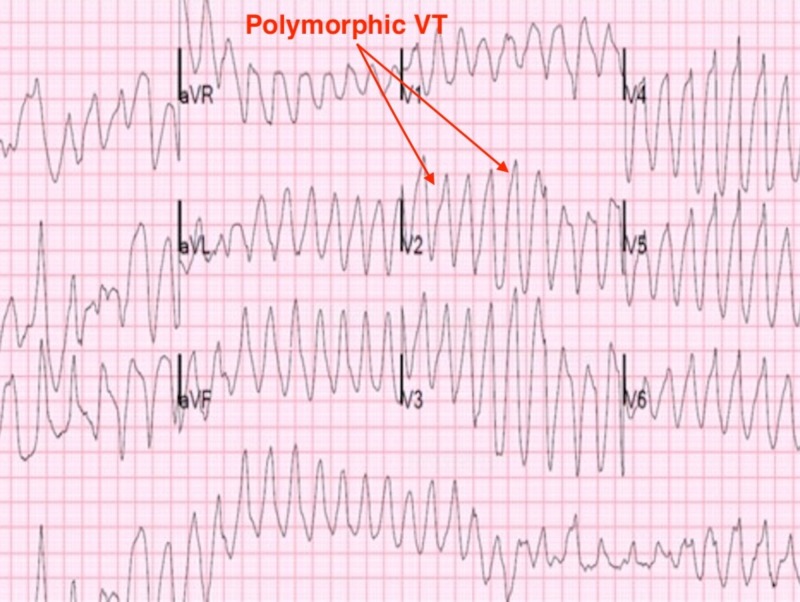
Electrocardiogram showing polymorphic ventricular tachycardia

A 43-year-old female patient with a past medical history of substance use disorder (SUD) was brought to the hospital by emergency medical services (EMS) presenting with multiple episodes of dizziness, shortness of breath, and palpitations. EMS reported ventricular tachycardia (VT) while in transit to the hospital for which she was externally defibrillated 10 times without any resolution of the ventricular arrhythmia. The patient’s past medical history was positive for depression and substance abuse of hydrocodone dating back to 2007. The patient’s husband denied any history of cardiac arrhythmias, structural heart disease, or ischemic heart disease. The patient’s husband denied using any medications, including herbal supplements, but reported drug allergies to amoxicillin and erythromycin. The patient’s social history revealed that she worked at a consignment store and had a history of alcohol and marijuana abuse. The patient’s husband also revealed recreational usage of loperamide at approximately 400 mg in the last 24 hours before the presentation. On physical exam in the emergency department, she appeared to be in moderate distress with pain from the external defibrillations. Her vital signs consisted of an elevated pulse of > 200 beats per minute (bpm), an elevated blood pressure of 157/80, and an increased respiratory rate at 24/minute. The pulmonary exam revealed decreased breath sounds bilaterally with symmetrical chest wall expansion. The cardiovascular exam revealed tachycardia with no murmurs reported. Examination of the head and neck showed moist mucous membranes with no jugular venous distension. The gastrointestinal exam was negative for distention, tenderness, rebound, guarding, hepatomegaly, or splenomegaly. The musculoskeletal exam revealed warm and well-perfused upper and lower extremities free of clubbing, cyanosis, and edema. In the processes of placing the electrocardiogram (EKG) electrodes onto the patient in the emergency room, the patient became unresponsive and the two lead EKG monitors showed polymorphic VT with a heart rate of 220 per minute and blood pressure of 84/58. The patient was immediately defibrillated once again before being sedated, intubated, and started on an amiodarone drip. The patient’s vitals were stabilized for 15 minutes before she went into another episode of polymorphic VT despite the amiodarone drip. The patient underwent synchronized cardioversion, and once her vitals stabilized, she underwent a cardiac catheterization which showed patent coronaries and a normal ejection fraction. The patient got transferred to the Medical Intensive Care Unit (MICU) for continuous monitoring and was started on a lidocaine drip in addition to the amiodarone drip. Her EKG in the MICU is shown below in Figure 1.

**Figure 2:**
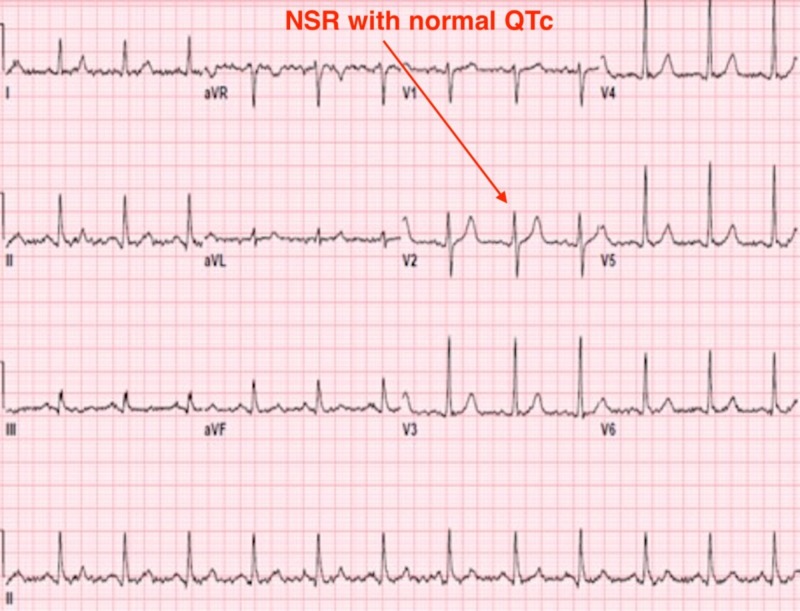
Electrocardiogram showing normal sinus rhythm (NSR) and normal QTc

In the MICU, the patient continued to have polymorphic VT episodes despite medical management and went in and out of VT episodes approximately 16 times with each episode lasting between 30 seconds to two minutes. Subsequently, the patient had a transvenous cardiac pacer placed which stabilized her vital signs. After 72 hours, the pacer was removed, and the patient remained stable on amiodarone and lidocaine. Transthoracic echocardiogram in the MICU revealed a normal ejection fraction with no valvular abnormality. Serum electrolytes were within normal limits, and the urine drug screen was also negative. After the temporary pacer was removed, the patient subsequently remained in sinus rhythm with a heart rate of 70 bpm, QRS duration of 98 ms, and a QTc interval of 456 ms as seen in Figure 2.

After five days of hospitalization, the effects of the loperamide had completely resolved, and the patient remained in normal sinus rhythm on amiodarone until discharge and followed up a month later.

## Discussion

The number of published case reports with regards to loperamide toxicity has been gradually increasing [1]. A total of 54 case reports have been published from 1985 to 2016, with 21 of those cases occurring between 1985 to 2013 and the remaining 33 cases occurring between 2014 and 2016. [1] Aside from published case reports, the National Poison Database System reports a total of 179 cases of intentional loperamide misuse between 2008 - 2016 with over half of those cases occurring after 2014 [1]. Consequently, the increasing trend in the misuse of the anti-diarrheal agent has shed light on its life-threatening cardiotoxic effects and arrhythmias, such as ventricular tachycardia storm (VTS). Ventricular tachycardia is a life-threatening cardiac arrhythmia brought on by those with structural heart disease, ischemic heart disease, and serum electrolyte abnormalities [2]. VTS is characterized by greater than or equal to three episodes of sustained VT, ventricular fibrillation (VF), or implantable cardioverter-defibrillator therapy within a 24-hour timeframe [3]. Loperamide is an anti-diarrheal agent that can be purchased over-the-counter and is commonly used and relatively inexpensive. The drug is a is a synthetic opiate that acts on peripheral µ opioid receptors primarily located within the gastrointestinal tract to decrease intestinal transit time and increase rectal tone [4]. Due to the drug's rapid metabolism and its inability of crossing the blood-brain barrier at therapeutic doses (2 - 16 mg), it is unable to precipitate the sought-after side effect of euphoria amongst substance abusers [5]. However, in recent years, substance abusers have exploited the drug's potent opioid agonizing properties by ingesting large doses (50 - 300 mg) of the drug to induce symptoms of euphoria [6-8]. Investigative studies have looked into loperamide’s inhibitory effects on L-type cardiac calcium channels which could, in turn, cause QT prolongation and lead to polymorphic VT [9]. However, the exact mechanism for loperamide’s cardiotoxicity associated with ventricular arrhythmias, QTc prolongation, and QRS prolongation are not clearly understood [10]. Our patient reported ingesting close to 400 mg per day and presented with sustained VT despite 10 attempts of external cardiac defibrillation. The uniqueness of our case is that our patient not only experienced one episode of VT but also had sustained and recurrent polymorphic VT with loperamide.

## Conclusions

Abrupt resolution of the VT, normalization of the patient's heart rate, QRS duration, and QTc interval without the need for an anti-arrhythmic after discontinuation of loperamide makes us believe a causal relationship exists, but the pathogenesis remains unclear. Future studies should focus on the cardiotoxic mechanisms associated with loperamide.
